# Resistin Response to Inflammation

**DOI:** 10.1371/journal.pmed.0010049

**Published:** 2004-11-30

Obesity, in particular visceral adiposity, is positively correlated with insulin resistance and type 2 diabetes. Although the link is well established in humans and in rodent models, the mechanisms involved in obesity-related insulin resistance are not clear. One possibility is that hormones secreted by adipocytes compromise peripheral insulin sensitivity, and a number of candidates for such adipocyte signals have been identified. One of them, resistin, was discovered a few years ago by Mitchell Lazar and colleagues, who showed that the protein is expressed by mouse adipocytes and regulated by a group of anti-diabetic drugs called thiazolidinediones. Several lines of evidence from functional studies in rodents suggested that resistin could be the missing mechanistic link between obesity and diabetes.

The human homolog of resistin has subsequently been under intense investigation, but initial studies revealed more differences than similarities between the human and rodent proteins: human resistin is mostly expressed in macrophages, not in adipocytes, and its serum levels do not correlate as clearly with obesity, insulin resistance, or diabetes. Similarly, genetic association studies between allelic variants of the resistin gene and metabolic abnormalities have so far been inconclusive. These results prompted some of the scientists in the field who had jumped on the resistin bandwagon after the initial results in rodents to jump off again. Others, including the resistin discoverers, continue their quest to uncover resistin's role in humans, and have started to think outside the framework defined by the mouse data.[Fig pmed-0010049-g001]

**Figure pmed-0010049-g001:**
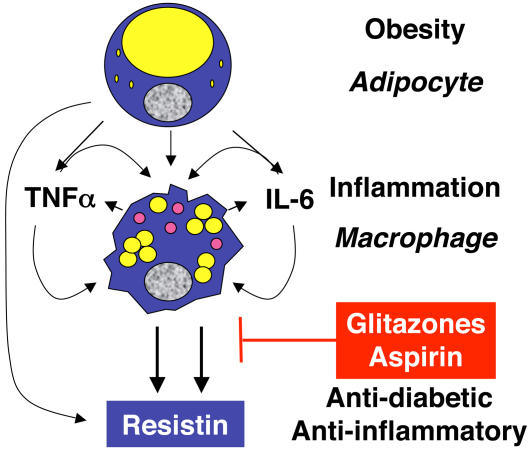
Connections between obesity and inflammation

Starting with the role of macrophages in inflammation and encouraged by the fact that obesity and insulin resistance are associated with markers of systemic inflammation, Lazar and colleagues examined the resistin response to inflammatory stimulators. As they report in this issue, resistin production in macrophages and serum levels in patients are significantly increased by these stimulators. This response can be blocked by the thiazolidinedione rosiglitazone and by aspirin, two drugs that have dual anti-inflammatory and insulin-sensitizing actions and antagonize the immune regulator NF-kappaB. The researchers go on to show that activation of NF-kappaB is sufficient to induce resistin expression. And NF-kappaB is necessary for the resistin response to inflammatory stimuli.

Lazar and colleagues now view obesity as a state of chronic inflammation and speculate that in obese individuals inflammatory cytokines lead to elevated production of resistin by macrophages and elevated serum resistin levels, which in turn contribute to insulin resistance and diabetes. This is consistent with some studies that have found higher resistin levels in obese individuals and patients with insulin resistance and/or diabetes, but not all studies have found such differences.

Jeffrey Flier, an obesity researcher who was not involved in the study, calls the article “an excellent and timely paper that demonstrates the fact that inflammatory pathways induce resistin expression and levels in human monocytes ex vivo, and in intact humans. The work appears to provide a novel link between inflammation and insulin resistance, through monocyte derived resistin.” He points out, however, that “several other factors also appear to contribute directly to insulin resistance in inflammation (e.g., cytokines themselves, without invoking resistin) so the full biologic implications of the high resistin levels for insulin resistance in humans cannot be determined from this study.” Resistin, it seems, continues to resist easy interpretations.

